# Advances in Technology-Assisted Visual Rehabilitation Therapies: Scoping Review

**DOI:** 10.1007/s10916-026-02410-4

**Published:** 2026-05-22

**Authors:** Mariana Saiz Briceño, Maria Paula Trujillo López, Laura Valentina Botia Suárez, Juan Camilo Tobo Hernández, Erwin Hernando Hernández Rincón

**Affiliations:** 1 Primary Care Physician, Universidad de La Sabana, Chía, Colombia; 2 Department of Family Medicine and Public Health, Universidad de La Sabana, Chía, Colombia; 3 Department of Family Medicine and Public Health, Universidad de La Sabana, Campus, Puente del común, km 7 Autopista Norte, Chía, 250001 Colombia

**Keywords:** Visual Rehabilitation, Visual Impairment, Artificial Intelligence, Telerehabilitation, Technology

## Abstract

Visual impairment (VI) is a growing condition associated with aging, neurological diseases, and chronic eye conditions. In 2023, more than 2.2 billion people experienced VI, and at least 1 billion could have been prevented or remain untreated. Visual rehabilitation (VR) is essential for optimizing residual vision and promoting functional independence; however, only a minority have access to these services. In recent years, the development of technologies such as artificial intelligence (AI), mobile applications, and virtual reality (VRt) has transformed VR, offering accessible and personalized alternatives. This scoping review aimed to map and categorize the available evidence on technology-assisted visual rehabilitation therapies, identifying the types of technologies used, their applications, and reported clinical and functional outcomes.

A scoping review was conducted following the Joanna Briggs Institute (JBI) methodology and PRISMA-ScR guidelines to map the literature published between 2015 and 2025 on technology-assisted VR therapies in adults with VI worldwide. Twenty-five studies, mainly from developed countries, were included. Interventions included videoconferencing, computerized systems, portable devices, VRt, artificial vision (AV), and smart sensors. Overall, digital technologies were shown to improve visual function, autonomy, and quality of life, with high patient satisfaction and adherence. Telerehabilitation (TR), understood as the remote delivery of visual rehabilitation services through videoconferencing and digital monitoring platforms, has emerged as a viable and cost-effective strategy. In contrast, immersive virtual reality technologies (VRt), head-mounted display systems, and multisensory integration devices have demonstrated potential to stimulate neuroplasticity and enhance functional visual outcomes.Telerehabilitation platforms, immersive virtual reality systems, portable electronic vision enhancement devices, artificial vision technologies, and sensor-based monitoring tools are emerging as promising interventions. These technologies have demonstrated improvements in visual acuity, reading performance, visual field sensitivity, patient adherence, and functional autonomy, although most studies remain small-scale and geographically concentrated in high-income settings.

## Introduction

Visual Impairment (VI) is an increasingly common condition that is largely explained by population growth, aging, and increased survival after serious brain diseases such as stroke or traumatic brain injury, as well as chronic eye diseases such as age-related macular degeneration, diabetic retinopathy, and advanced glaucoma [1]Globally, in 2023, there will be 2.2 billion people with VI. In at least 1 billion of these cases, VI could have been prevented or has not yet been treated [1]. In addition, 73% of those seeking outpatient Visual Rehabilitation (VR) are older adults [2]. However, access to these services remains limited due to shortages of specialized professionals, economic and mobility barriers, and inequitable distribution of services; it is estimated that only 3%–15% of the population who could benefit from VR access it [3].

VR therapy is a set of medical, educational, and social interventions aimed at improving visual function and reducing the negative impact of VI [3]. Its objective is to optimize residual vision and promote functional independence, facilitating the patient’s adaptation to their environment. These interventions include visual skills training; the use of optical and non-optical aids; and orientation, mobility, and psychosocial support strategies [4]. For these reasons, it is recognized as an essential component in the comprehensive management of people with VI, especially when medical or surgical treatments fail to fully restore visual function [5].

In recent years, technology has revolutionized this field, especially in developed countries. Noteworthy examples include Telerehabilitation (TR) delivered through videoconferencing platforms [6–8], portable audiovisual and multisensory devices [9–12],], and digital tools such as Artificial Intelligence (AI), mobile applications, and Virtual Reality Technologies (VRt) [13, 14] have enabled more accessible, personalized, and potentially cost-effective interventions [15]. These digital approaches are increasingly recognized as complementary components of primary health care strategies aimed at disability prevention, social inclusion, and healthy ageing [3].

Given the rapid technological evolution and heterogeneity of interventions, this research is appropriate to comprehensively map existing evidence, identify knowledge gaps, and clarify the range of technologies currently used in VR for people with VI.

Thus, the aim of this research is to map and categorize the available evidence on technology-assisted visual rehabilitation therapies for adults with visual impairment, describing the types of technologies used, their clinical applications, reported outcomes, and identified benefits and limitations.

## Materials and Methods

A scoping review was conducted in October 2025, following the Joanna Briggs Institute (JBI) methodology and PRISMA-ScR guidelines [16, 17], and the protocol was registered on the OSF platform. The research question guiding this review is as follows: What is the available evidence on the use of technology in visual rehabilitation therapies for people with visual impairment?

The inclusion criteria were structured using the PCC (Population, Concept, Context) framework.

- Population: Adults (≥ 18 years) with visual impairment of any etiology.
- Concept: Technology-assisted visual rehabilitation interventions, including digital, electronic, sensor-based, immersive, or AI-supported tools.
- Context: Clinical, home-based, or community-based rehabilitation settings worldwide.

Studies focusing exclusively on surgical techniques, pharmacological treatments, or non-technological conventional rehabilitation were excluded.

### Search strategy

The search was conducted in three scientific databases, PubMed, Scopus, and BIREME, where articles related to the research question were identified. Studies published between January 2015, and October 2025 were included to capture contemporary digital and technology-assisted interventions, given the rapid evolution of virtual reality, artificial intelligence, and mobile-based rehabilitation tools in the past decade. Earlier studies were excluded because many technologies evaluated prior to 2015 are no longer representative of current digital rehabilitation platforms. Articles in English and Spanish were included. The search terms were designed using MeSH-DeCS descriptors and related keywords.

The specific search strategies for each database, as well as the inclusion and exclusion criteria applied, are detailed in Table 1. These strategies were adapted to the particularities of each database to ensure broad and accurate coverage of the relevant literature. PubMed was included for its biomedical focus and broad coverage, Scopus for its interdisciplinary scope, and BIREME for its access to the literature from Latin America and the Caribbean.

**Table 1:** Search strategies and inclusion and exclusion criteria

Inclusion criteria	• Studies that evaluate, describe, or implement technologies applied to visual rehabilitation in people with visual impairments. • Types of technologies: virtual reality, augmented reality, artificial intelligence, visual training software or video games, electronic devices, eye stimulation or tracking systems. • Publications between January 2015 and October 2025. • Articles in English or Spanish. • Scientific articles, systematic or scoping reviews, qualitative, quantitative, or mixed studies, and technical reports. • Adult patients over 18 years old.	
**Exclusion criteria**	• Editorials, letters to the editor, opinions without empirical support, narrative reviews, case reports. • Studies published outside the established time frame. • Studies focused exclusively on prevention, diagnosis, or medical/surgical treatment of eye diseases, without a rehabilitation component. • Publications focused solely on clinical or surgical interventions, unrelated to the use of technologies. • Publications without access to the full text or without sufficient methodological description.	
**Search strategies**	**PubMed**	((“visual rehabilitation“[tiab] OR “vision rehabilitation“[tiab] OR “visual training“[tiab] OR “vision therapy“[tiab]) AND (“visual impairment“[tiab] OR “low vision“[tiab] OR blindness[tiab]) AND (“virtual reality“[tiab] OR “augmented reality“[tiab] OR “artificial intelligence“[tiab] OR “machine learning“[tiab] OR “deep learning“[tiab] OR “eye tracking“[tiab] OR “video game*“[tiab] OR gamification[tiab] OR software[tiab] OR app[tiab] OR wearable*[tiab] OR mhealth[tiab] OR ehealth[tiab] OR “assistive technology“[tiab] OR “digital technology“[tiab])) AND (“2015/01/01“[Date - Publication] : “2025/10/31“[Date - Publication]) AND (english[lang] OR spanish[lang]) NOT (editorial[pt] OR letter[pt] OR comment[pt] OR “case reports“[pt] OR news[pt])
	**Scopus**	(TITLE-ABS-KEY(“visual rehabilitation” OR “vision rehabilitation” OR “visual training” OR “vision therapy”) AND TITLE-ABS-KEY(“visual impairment” OR “low vision” OR blindness) AND TITLE-ABS-KEY(“virtual reality” OR “augmented reality” OR “artificial intelligence” OR “machine learning” OR “deep learning” OR “eye tracking” OR “video game*” OR “serious game*” OR gamif* OR software OR app* OR wearable* OR mhealth OR ehealth OR “assistive technology” OR “digital technology” OR “head-mounted display*” OR HMD) AND TITLE-ABS-KEY(adult* OR “older adults”))
	**BIREME (LILACS + SciELO)**	((“rehabilitación visual” OR “rehabilitación de la visión” OR “entrenamiento visual” OR “terapia visual” OR “visual rehabilitation” OR “vision rehabilitation” OR “low vision rehabilitation” OR “visual training” OR “vision therapy”) AND (“discapacidad visual” OR “baja visión” OR “deficiencia visual” OR ceguera OR “visual impairment” OR “low vision” OR “visual disability” OR blindness) AND (“realidad virtual” OR “realidad aumentada” OR “realidad mixta” OR “inteligencia artificial” OR “aprendizaje automático” OR “machine learning” OR “deep learning” OR “seguimiento ocular” OR “eye tracking” OR “estimulación visual” OR “videojuego*” OR “juego serio” OR gamificación OR software OR aplicación OR app* OR dispositivo* OR “tecnología electrónica” OR “salud móvil” OR mhealth OR ehealth OR “tecnología asistiva” OR “tecnología digital”)) AND (adult* OR adulto* OR “mayores de 18 años” OR “personas adultas” OR “older adults”)

### Study Selection

The citations were imported into the Rayyan platform, designed for data management, duplicate articles were removed, and four reviewers (Mariana Saiz Briceño, Maria Paula Trujillo Lopez, Juan Camilo Tobo Hernandez and Laura Valentina Botia Suarez) independently selected titles and abstracts according to the inclusion and exclusion criteria. Any discrepancies were resolved jointly by the four reviewers to reach a final agreement. Finally, the four reviewers conducted an in-depth reading of the selected articles to determine the final eligibility of the studies.

### Data Extraction

Data extraction was performed via a table previously designed by the research team. This table included the following items: article name, year of publication, authors, journal of publication, country or location, type of study, study objective, population size or number of articles per study, type of VI, key results and findings, type of technology used, application of technology, advantages of the technology applied, limitations of the technology applied, future opportunities, and ethical considerations. The data collection was carried out independently by four reviewers, and any discrepancies were resolved by consensus.

### Data Analysis and Synthesis

The collected data were analysed via narrative and descriptive synthesis, and the information was organized according to their primary functional purpose. A quantitative and qualitative comparison of the different approaches was subsequently carried out to identify similarities and differences in their implementation. Finally, the advantages, limitations, and future opportunities described in the literature were integrated for the development, optimization, and validation of these technologies.

## Results

**Fig. 1:**
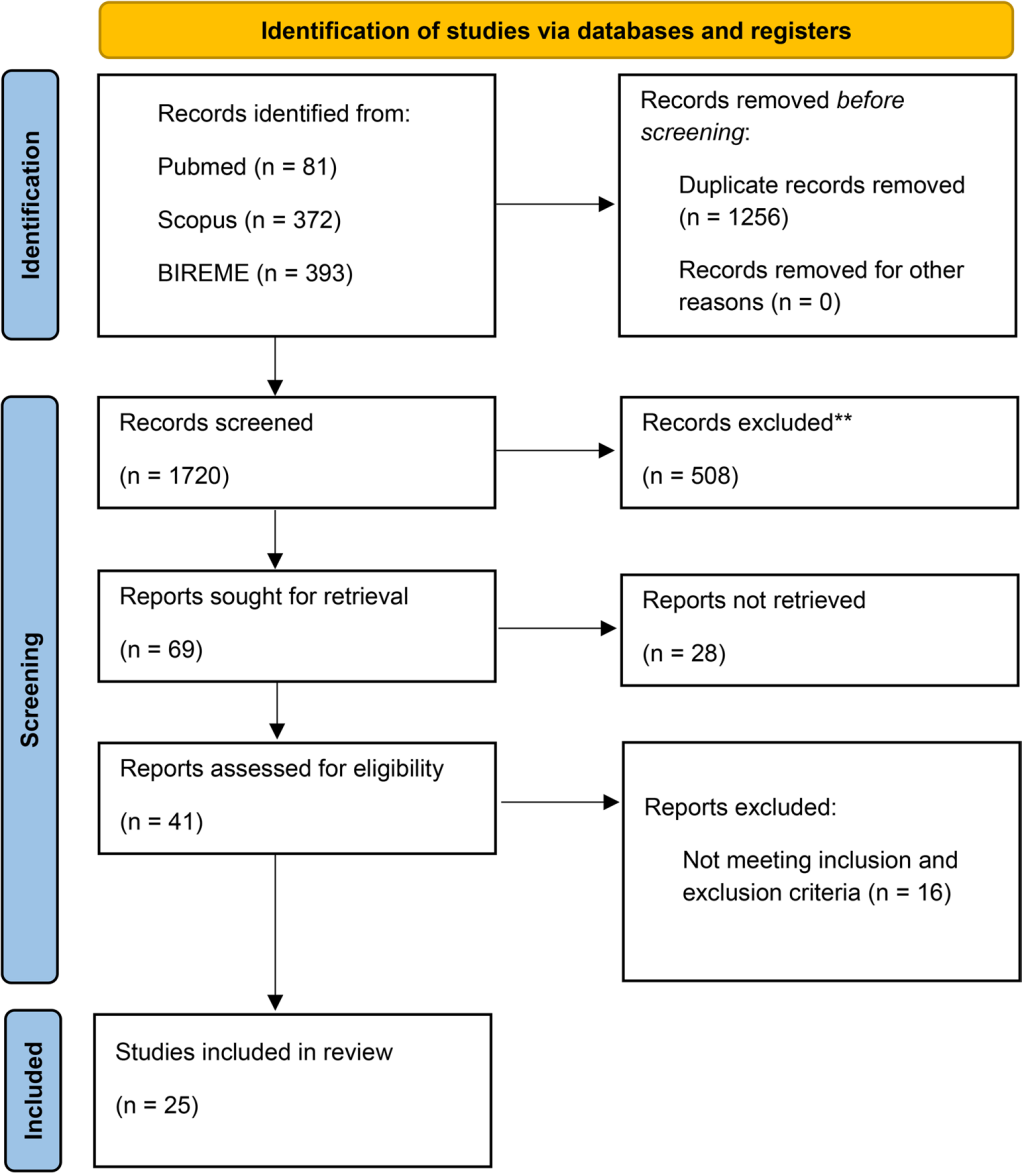
Prisma flowchart

A total of 846 articles were identified in the initial search. Of these, 269 (31.8%) were eliminated because they were duplicates. A total of 577 (68.2%) studies were reviewed, of which 508 were excluded after reviewing the title and abstract because they did not meet the inclusion criteria. Sixty-nine articles were selected for full-text reading, twenty-eight articles could not be accessed despite institutional database searches and interlibrary loan requests; most were unavailable due to subscription restrictions, inaccessible full texts, or incomplete archival records, and 16 articles did not meet the inclusion criteria. In total, 25 studies were included in this review. The selection and flow of the included articles are presented via the PRISMA model in Fig. 1.

### Data Characteristics

Twenty-five articles evaluating technologies applied to VR were included. Among them, randomized clinical trials (*n* = 6), including controlled, double-blind, and multicentre designs, focused mainly on evaluating the effectiveness of technological interventions, were identified. Most were nonrandomized experimental studies (*n* = 9), with pilot, comparative, multicentre, and training/testing designs. There were also observational studies (*n* = 1), experimental pilot studies (*n* = 2), non-experimental prospective studies (*n* = 4), quasi-experimental studies (*n* = 1), and descriptive/qualitative studies (*n* = 2), including one cross-sectional design and one descriptive qualitative design.

**Fig. 2:**
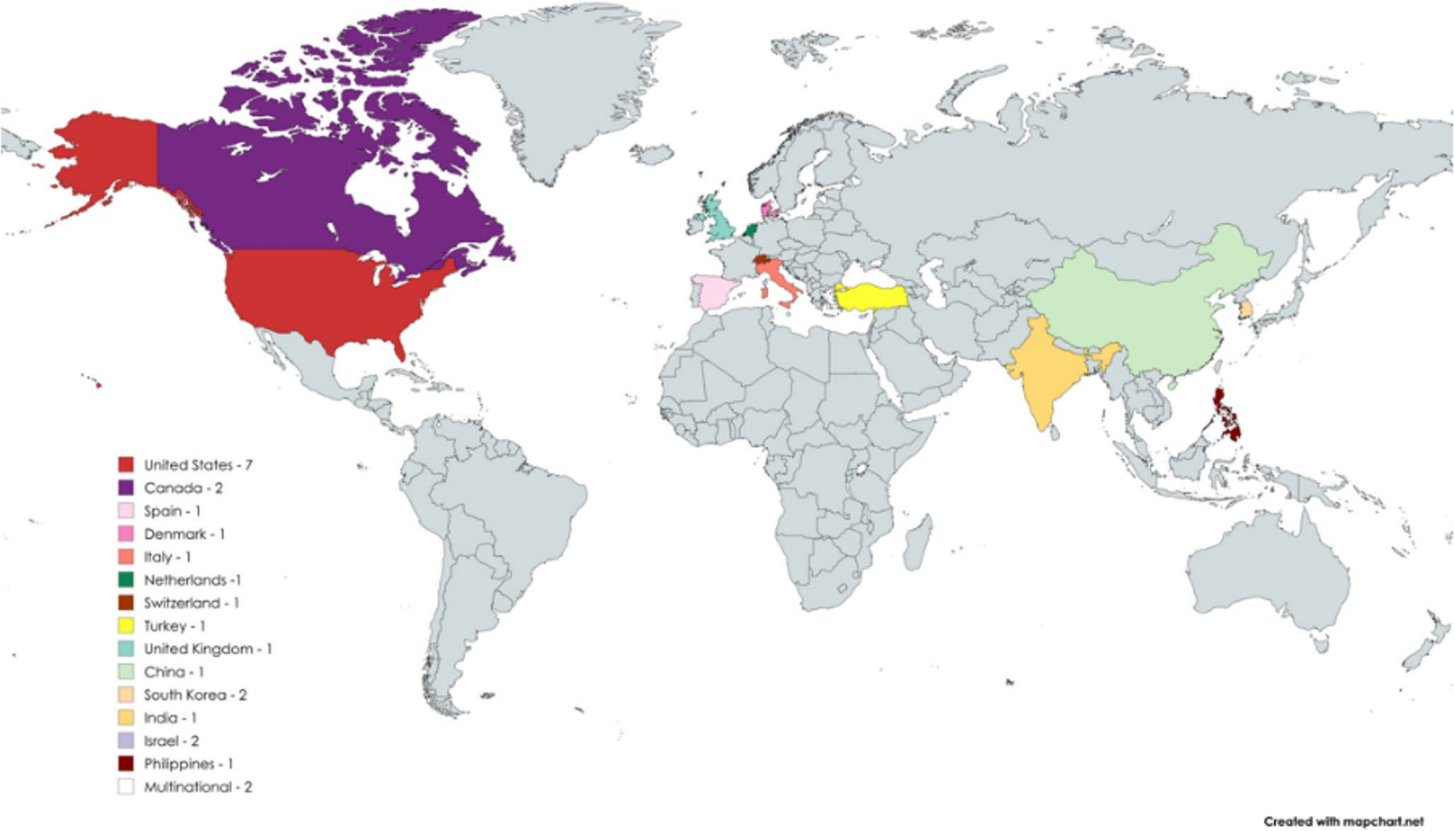
Geographic distribution of studies

In terms of temporal distribution, the included studies were published between 2015 and 2025. During the first period (2015–2019), the evidence was limited (*n* = 8). From 2020 onwards, there was a sustained increase in the number of publications, with 2020 (*n* = 1), 2021 (*n* = 4), and 2022 (*n* = 4) representing the highest points of productivity in the period analysed. In recent years, production has remained stable, with a slight decrease (2023 *n* = 3; 2024 *n* = 3; 2025 *n* = 2). The geographical distribution of the studies, classified by continent, is illustrated in Fig. 2.

The technologies used in the studies were Artificial Vision (AV) and VRt devices (*n* = 6), followed by portable audiovisual rehabilitation devices (*n* = 5), electronic devices (*n* = 4), and head-mounted devices (*n* = 4). To a lesser extent, computerized systems and specialized software (*n* = 2), videoconferencing-based technologies (*n* = 2), Bluetooth-enabled sensors (*n* = 1), and retinal prostheses (*n* = 1) were identified.

### 1. Remote delivery and monitoring technologies (telerehabilitation and sensors) 

#### Videoconferencing Technologies

Two studies on videoconference-based interventions were analysed [6, 18], which demonstrated that TR is an effective and accepted alternative to face-to-face models for people with low vision. The participants showed comparable improvements in visual function and quality of life, with high rates of satisfaction and adherence (83–94%). The remote modality reduced travel costs and waiting times, encouraging participation by older adults and even users with no digital experience. 91% of patients felt comfortable with virtual sessions, and 78% reported improvements in the use of magnifiers [6]. Additionally, remote training reinforces practice, confidence, and motivation, facilitating the correct use of devices and compensating for the limitations of in-person attendance [18]. Overall, videoconferencing is establishing itself as a viable, safe, and cost-effective tool for home VR, if it is accompanied by technological support and basic training.

#### Bluetooth Technology Sensors

A study using low-energy Bluetooth technology [19] was analysed, demonstrating the feasibility of using portable sensors to objectively and continuously monitor the home use of visual aids in people with low vision. Sensors installed in handheld optical magnifiers reliably detected periods of activity through variations in movement and temperature, showing a significant correlation with self-reported records. This approach made it possible to quantify the actual frequency and duration of device use, providing an accurate indicator of VR adherence and effectiveness. In addition, the system is inexpensive, non-invasive, and easily integrated into TR or remote monitoring programs, representing a step forward in the intelligent monitoring of visual behaviour at home.

### 2. Reading and near-vision support applications

#### Computerized Systems and Specialized Software

Two studies evaluating computerized systems and specialized software were analysed. A prospective, observational experimental study applied software-assisted training with eye tracking and structural magnetic resonance imaging, showing improvements in the extension and sensitivity of the Visual Field (VF), although with no changes in the optic tract; its degeneration was identified as a predictor of recovery, and the importance of early intervention was highlighted [7]. Additionally, a randomized controlled crossover trial used a home-based platform with visual discrimination tasks and webcam fixation control, reporting improvements in perimetric performance and reading fluency, with localized effects and individual variability [8]. Therefore, the use of computerized systems as intensive, customizable, and home-applicable tools is supported, although it is limited by sample size, response heterogeneity, and calibration and fixation challenges.

### Electronic Devices

Four studies evaluating the use of electronic devices were analysed. The iPad was explored as a support tool for people with low vision showing improvements in independence, social connectivity, and self-esteem, owing to accessibility features such as magnification, voice reading, and communication via FaceTime [20]. In addition, the printed version and the digital application of the MNREAD reading test were compared, finding equivalence in Visual Acuity (VA) and reading accessibility, which supports the use of the tablet version as a practical and reliable alternative in clinical settings [21]. Similarly, an experimental study [22] showed that reading performance with an iPad was comparable to that with a portable video amplifier, with high satisfaction in terms of ease of use and effectiveness. Finally, a descriptive study [23] on mobile applications with accessibility features revealed that they improve functional independence and digital inclusion, especially in reading, navigation, and communication tasks. Therefore, electronic devices stand out as accessible, multifunctional, and socially integrative tools capable of promoting the autonomy and participation of people with VI.

### 3. Multisensory substitution and integration systems. 

#### Portable Audiovisual Rehabilitation Devices

Five articles that used portable audiovisual devices were analysed, and these technologies promoted VR through multisensory feedback and portable electronic assistance. Visual-auditory sensory substitution systems, such as EyeMusic [11, 24], enable people with congenital blindness to learn to recognize and integrate complex shapes, such as faces and emotions, from sound stimuli, demonstrating CP and perceptual learning even without prior visual experience.

Similarly, Audiovisual Thumble [10] significantly improved auditory and visual localization in patients with macular degeneration, confirming the value of combined feedback (auditory, visual, and proprioceptive) in enhancing spatial orientation. In patients with acquired brain damage, computer-controlled oculomotor training [9] improved the accuracy and speed of eye movements, demonstrating functional adaptations after therapy. Finally, the use of the portable eSight [12] in people with age-related macular degeneration produced quantifiable improvements in VA and quality of life, facilitating reading, facial recognition, and daily mobility. Taken together, these findings demonstrate that portable audiovisual devices represent promising tools for VR, combining sensory stimulation, interactive training, and functional assistance, and promoting independence and CP in different types of visual loss.

### 4. Visual function enhancement technologies.

#### Artificial Vision and Virtual Reality Devices

Six studies evaluating AV devices and VRt were analysed. The use of the BrainPort device [25] demonstrated that intensive training enabled people with total blindness to identify objects and words through tactile stimulation. The smartphone-based Relumino system [26] improved VA, facial recognition, and quality of life, with high satisfaction and good tolerance. Similarly, Nunap Vision digital therapy [27] has shown clinical improvements in post-stroke VF defects and a favourable safety profile. Other trials with head-mounted VRt systems [28–30] reported significant increases in VF sensitivity, greater fixation stability, improved functional perception, and quality of life compared with conventional therapy. Therefore, these studies support the efficacy and safety of these devices as effective, safe, and motivating strategies to promote visual recovery and Cortical Plasticity (CP) with varying degrees of VF loss.

### Retinal Prosthesis 

A study on retinal prostheses [31] evaluated visual cortical function in people with blindness due to retinitis pigmentosa who had received the Argus II implant and compared it with that of controls with simulated reduced vision. The results revealed that Argus II users had Visual Evoked Potentials (VEP) with morphologies like normal vision, suggesting functional preservation of the early visual cortex even after years of blindness. Although there was individual variability in latency and amplitude, most participants generated reproducible signals, indicating that effective visual processing was induced by electrical stimulation. These findings confirm that VEP are an objective tool for evaluating and adjusting retinal prostheses and support their potential to partially restore visual function in cases of severe loss.

### Head-Mounted Devices

Four studies evaluating head-mounted devices were analysed. The use of electronic viewers such as AceSight, eSight, IrisVision, and Jordy in patients with Stargardt’s disease resulted in improvements in VA, contrast, and reading, although prolonged use was limited by fatigue and social factors [32]. These aids have also been shown to optimize reading, facial recognition, and quality of life in people with moderate to severe low vision [33]. Furthermore, transcranial direct current stimulation applied to the visual cortex increased VA and processing speed in patients with proliferative diabetic retinopathy [34], whereas its combination with visual training and non-invasive electrical stimulation accelerated perceptual learning and maintained functional improvements in cases of cortical blindness [35]. Therefore, head-mounted devices and visual stimulation techniques are effective, safe, and adaptable tools capable of enhancing visual function and CP in different types of VI.

### Benefits and Limitations

The benefits and limitations of technology in VR therapies for people with a VI extracted from the articles evaluated are presented in Table 2.

**Table 2:** Benefits and limitations of technology in visual rehabilitation therapies for people with visual impairments

Category	Description	References
**Benefits**	Improvement in visual acuity, contrast sensitivity, visual field sensitivity, and reading speed.	[12, 32–34]
	Increase functional independence, quality of life, and personal autonomy.	[19, 22, 33]
	High satisfaction, adherence, and motivation among participants, particularly during home-based or remote training programs.	[8, 18, 25, 27]
	Affordability, portability, and reduction of geographical barriers, facilitating continuity of rehabilitation.	[6, 18, 19, 21]
	Customization of visual parameters and compatibility with daily activities, enhancing user experience.	[12, 24]
	Evidence of cortical plasticity and sustained perceptual learning, supporting the neurorehabilitative potential of these technologies.	[7, 9, 28, 35]
**Limitations**	Small sample sizes and lack of control groups, reducing external validity, and limiting the strength of conclusions regarding the effectiveness of interventions.	[7, 9, 10, 19, 25–27, 32–35]
	Short follow-up periods, with no longitudinal assessments to determine the persistence or generalization of therapeutic effects.	[8, 19, 26–28]
	Visual fatigue, device weight, and discomfort during prolonged use, as well as social or aesthetic rejection due to size or visibility.	[12, 24, 32, 33]
	Technical and calibration issues, including disconnections, data loss, and variability in ocular fixation, compromising the consistency of results.	[8, 18, 30]
	Scarcity of functional and objective outcome measures, along with the absence of neuroimaging or neurophysiological correlates, hindering the understanding of behavioral improvements in relation to neural mechanisms.	[7, 9, 19, 34, 35]

## Discussion

### Synthesis of Results

This review synthesizes contemporary evidence on technology-assisted visual rehabilitation and highlights a shift from device-centered approaches to function-oriented, personalized interventions. Rather than merely introducing new tools, these technologies appear to modify therapeutic delivery models, enhance cortical plasticity mechanisms, and expand access beyond traditional clinical environments. This pradigm shift is consistent with previous literature, which has increasingly emphasized patient-centered rehabilitation strategies.

Video conferencing interventions improve therapeutic accessibility, demonstrating that TR offers results like those of face-to-face therapy. In the studies by Bittner et al., remote programs achieved 83–94% satisfaction and 78–91% adherence, even in older adults or those without technological experience. The participants demonstrated functional improvements and greater confidence in the use of magnifiers, reading and mobility. Platforms such as Zoom allowed for continuous sessions, reduced costs and travel, and confirmed their effectiveness as an extension of the face-to-face model. These findings align with prior reports on tele-rehabilitation in other clinical fields, where accessibility and adherence are consistently improved without compromising clinical outcomes.

Computerised systems and specialised software yielded consistent results. An experimental study used an eye-tracking training programme and demonstrated that early intervention improved the sensitivity and extent of VF. Another home-based platform with webcam-controlled visual discrimination tasks allowed for automatic difficulty adjustment and adherence tracking. This approach was associated with improvements in perimetric performance and reading fluency. Moreover, the use of electronic devices, such as iPads and mobile applications, has become established as a convenient and multifunctional alternative for VR. Their increasing accessibility, affordability, and widespread adoption make them a particularly suitable option for expanding rehabilitation beyond traditional clinical settings. Mendik et al. reported improvements in independence, self-esteem, and social connectivity through accessibility features, including voice reading, magnification, and communication tools. Wittich et al. demonstrated that the iPad offers performance comparable to that of portable video magnifiers, with high satisfaction due to its ease of use, consolidating these devices as cost-effective and socially integrative options for promoting autonomy in different degrees of visual loss.

Among advanced technologies, AV devices and therapeutic VRt systems have a significant impact on visual function and cortical reorganisation. BrainPort, which converts visual information into lingual tactile stimulation, enables people with total blindness to recognise objects and words, demonstrating CP. The smartphone-based Relumino system improved VA, facial recognition and quality of life, whereas Nunap Vision digital therapy proved effective and safe in postictus VF defects. Head-mounted VRt systems increased VF sensitivity and fixation stability, with perceptual and quality -of -life improvements superior to those of conventional treatments. These findings confirm that VRt activates residual visual areas through repetitive and immersive stimulation, promoting functional reorganisation of the visual cortex.

Portable audiovisual rehabilitation devices (EyeMusic, Audiovisual Thumble, eSight) integrate visual, auditory, and proprioceptive feedback to enhance spatial perception and recognition of complex stimuli. EyeMusic enables people with congenital blindness to learn to identify shapes and faces through sound patterns, demonstrating perceptual learning without prior visual experience. Audiovisual thrombosis improved auditory and visual localisation in macular degeneration, whereas computer-assisted oculomotor training optimised the accuracy and speed of eye movements in acquired brain injury. These technologies confirm that multisensory integration and adaptive training strengthen CP and promote functional recovery.

Bluetooth-enabled sensors allowed for objective quantification of the frequency and duration of home use of optical aids, which was correlated with self-reported records. This inexpensive and accurate system was easily integrated into TR programs as an indicator of therapeutic adherence. In retinal prostheses, the Argus II implant presented VEP with a morphology like that of normal vision, suggesting functional preservation of the early visual cortex. Head-mounted devices (AceSight, IrisVision, Jordy, and eSight) improve VA, contrast, and reading in hereditary diseases and severe low vision. Similarly, transcranial direct current stimulation (tDCS) combined with noninvasive electrical stimulation increased processing speed and perceptual learning in patients with diabetic retinopathy and cortical blindness. Although prolonged use may be limited by visual fatigue or social factors, its benefits confirm the ability of brain stimulation to modulate CP and enhance conventional VR. Recently, AI has begun to play a prominent role in VR, demonstrating advances in accessibility, autonomy, and functional performance [36]. This growing role of AI reflects broader trends reported in the literature, where intelligent systems are increasingly integrated into personalized rehabilitation pathways, not only in visual rehabilitation but also across diverse rehabilitation fields, underscoring its potential as a scalable and transformative approach for future patient-centered care [37].

**Fig. 3:**
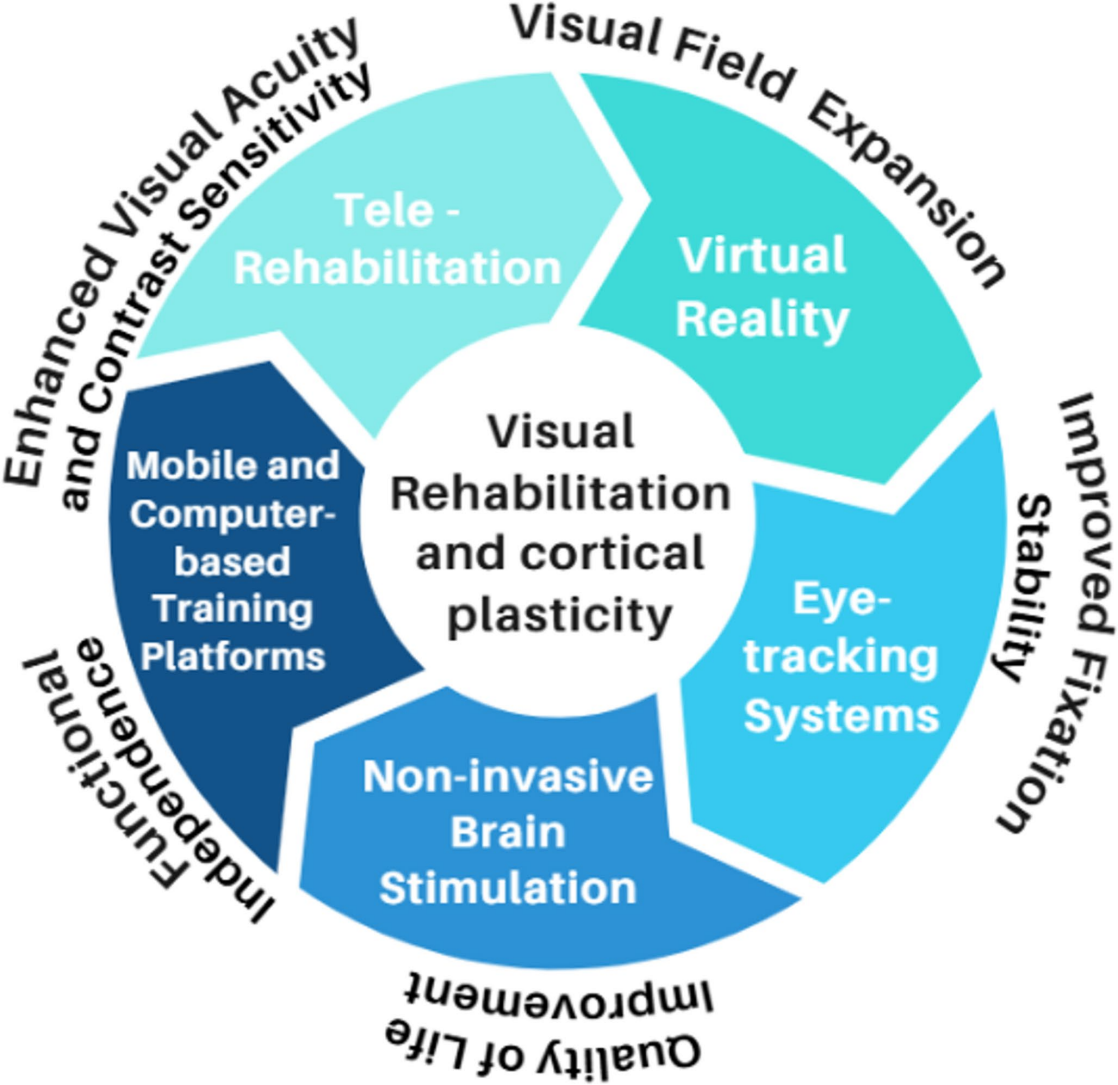
Integrative circuit of technologies in visual rehabilitation and neurofunctional mechanisms

As illustrated in Fig. 3, all these tools converge in an integrative circuit where technological stimulation, neurofunctional mechanisms, and clinical outcomes form a continuum of CP and functional recovery through repetitive, multisensory, and personalised stimulation. These findings consolidate technology-assisted VR as a comprehensive approach capable of expanding access, promoting independence, and ensuring therapeutic continuity. Overall, our findings are consistent with the current body of literature, while also extending it by integrating diverse technological modalities into a unified conceptual framework of visual rehabilitation.

### Global Equity and Gaps

Evidence on technology-assisted VR therapies comes almost exclusively from high-income countries such as the United States, Canada, the United Kingdom, Germany, and Italy, with little representation from regions such as Asia and a notable absence of Latin American studies. This concentration highlights geographical and technological equity gaps, related not only to the availability of resources but also to structural barriers such as the cost of devices, digital connectivity, technological literacy, and the lack of public policies that promote digital inclusion in visual health. In this context, visual TR is emerging as a promising alternative for reducing inequalities and expanding access in rural or hard-to-reach areas by facilitating remote monitoring and therapeutic continuity, thus offering a cost-effective and equitable way to improve visual care in low- and middle-income countries.

### Clinical Relevance

Technology-assisted VR represents a significant clinical advance, as it facilitates functional recovery and autonomy for people with visual loss through digital, portable, and remotely accessible tools. These interventions not only reduce geographical and economic barriers, but also enable more continuous, personalized and evidence-based care [25]. Conducting this scoping review is of great clinical value, as it allows us to map the most effective technologies, understand their real-world applications in different contexts, and guide future research and policies to safely and equitably integrate technological innovation into VR practice.

### Future Opportunities

The literature highlights opportunities to strengthen the application of technologies in VR. Multicentre studies with large, heterogeneous samples including different age groups and levels of visual loss are needed to validate the results and improve their generalizability [9, 20, 28–30, 34]. It has also been proposed to incorporate AI and augmented VRt to personalize programs and automate the analysis of results [12, 18, 19, 26], in addition to optimizing the design and ergonomics of devices together with TR strategies and home training that improve accessibility [10, 18, 22, 32]. CP should be further explored through neuroimaging studies, and standardized protocols and shared databases should be established to consolidate clinical evidence [7, 31, 34, 35]. It is also a priority to validate tools in Latin American countries, including diverse samples, and evaluate long-term functional outcomes, strengthening the applicability and equity of interventions. Overall, future projections point towards more personalized, accessible, and evidence-based care, driven by technological integration and collaborative research.

#### Ethical Considerations

All studies included in this review complied with the ethical principles set out in the Declaration of Helsinki and were approved by institutional or research ethics committees. In most cases, written or verbal informed consent was obtained from the participants. No relevant conflicts of interest or ethical violations were identified in the studies reviewed. This review was based exclusively on previously published and publicly available literature and therefore did not require approval by an ethics committee or additional informed consent.

### Limitations

This review has several limitations. First, the heterogeneity in study designs, intervention protocols, outcome measures, and definitions of visual impairment limited direct comparison across studies. Second, the search was restricted to English and Spanish publications and three databases, which may have resulted in the exclusion of relevant studies from other linguistic or regional contexts. Third, many included studies were pilot or small-sample trials conducted in controlled environments, which may affect external validity. Finally, cost-effectiveness analyses and direct comparisons between in-person and telerehabilitation models were limited in the available literature.

## Conclusion

The evidence indicates that telerehabilitation platforms, immersive virtual reality systems, portable electronic vision enhancement devices, artificial vision technologies, and sensor-based monitoring tools are emerging as promising interventions in visual rehabilitation. These technologies demonstrate improvements in visual acuity, visual field sensitivity, reading performance, autonomy, adherence, and patient satisfaction. However, most evidence derives from small or single-centre studies in high-income countries. Future multicentre and longitudinal research, including direct comparisons between in-person and remote models, is required to strengthen the evidence base and ensure equitable, scalable, and ethically sustainable implementation.

## Data Availability

The complete data extraction table used in this review is available in an open repository (Zenodo, [38].)

## Abbreviations

VI: Visual Impairment
VR: Visual Rehabilitation
AI: Artificial Intelligence
VF: Visual Field
AV: Artificial Vision
VA: Visual Acuity
VEP: Visual Evoked Potentials
VRt: Virtual Reality Technologies
TR: Telerehabilitation
CP: Cortical Plasticity
